# New Method of Arresting Epistaxis

**Published:** 1872-10

**Authors:** 


### ARTICLE XI.

### *New Method of Arresting Epistaxis.*

Dr. F. A. Marin, of Geneva (*Journal de Med. et de Chir-Prat.*, May 1872,) has discovered a new and simple method of arresting hæmorrhage from the nasal cavity. It consists in applying pressure to the facial artery at a point immediately beneath the ala of the nose, where the vessel can be pressed against the maxillary bone. In epistaxis the hæmor rhage is usually confined to the anterior third of one of the nasal fossæ, and as a pressure upon the facial artery causes a diminution in the flow of blood to this cavity, the hæmor. rhage is arrested almost immediately, and this proceeding is therefore recommended as preferable to that of plugging the posterior nares by the aid of Belloc's sound, in attempting which the surgeon is generally pretty thoroughly smeared with blood, and is not unfrequently bitten. Dr. Marin has had occasion to give numerous trials to this method suggested by him, and as generally found it effective. In two instances where it failed, plugging the posterior nares was attended with like result.—*Boston Medical and Surgical Journal.*

